# Improving Diabetes Care: A Fijian Diabetes Service Improvement Study

**DOI:** 10.1155/2022/9486679

**Published:** 2022-04-13

**Authors:** Abdul Mushib Ibrahim, Stephen Lawrence

**Affiliations:** ^1^Department of Emergency Medicine, Colonial War Memorial Hospital, Ministry of Health and Medical Services, MSc-Diabetes, MBBS-Labasa Divisional Hospital, Fiji; ^2^Principal Clinical Teaching Fellow, University of Warwick, UK

## Abstract

**Background:**

Achieving good outcomes in type 2 diabetes mellitus patients' needs a decent integrated care service with access to resources. The Fiji Islands has one of the highest rates of diabetes disease burden and has available resources to alleviate the diabetic disease pandemic in its population, yet patient outcomes are getting worse. We hypothesize that a dysfunction in health-care delivery system may be accentuating the diabetic disease process; therefore, this service evaluation study was conducted to provide insight into the management of T2DM in a secondary care clinic setting.

**Methods:**

We conducted a retrospective chart review of patient records for the past three years (2015-2018). Random quota sampling was used to extract patient folders over a one-month period. A total of 113 patient charts were analyzed which met the inclusion criteria.

**Results:**

The overall glycemic levels were uncontrolled in every seven out of ten patients. Most of the patients were on combination drug therapy and at maximum dosing ranges. HbA1c tests, as a monitoring tool, were being inadequately used. Nonadherence to management was prominent in poor controlled patients, and physicians failed to provide appropriate interventions in this group. Nearly all the patients had not received eye assessments, foot risk assessment, and individualized dietetic counselling over three years. Macrovascular complications were more common than microvascular end organ damage.

**Conclusion:**

There is a high degree of uncontrolled glycemia and comorbidities in patients attending the service of study which is being perpetuated by poor integrated diabetes care. Strengthening educational initiatives, using validated strategic tools to streamline diabetic services and astute evidence-based resource allocation and management, is needed.

## 1. Background

Type 2 diabetes mellitus (T2DM) is known to be one of the most debilitating chronic diseases of the modern era that results in substantial burden to human, social, and economic costs and also strains nearly all health-care systems [1]. Current data available states that people living with diabetes at a global level stand at 425 million as of 2017, and estimated projections suggests that there will be an increase by 48% to 629 million by 2045 [2].

The Fiji Islands, being a small island, middle-income nation with a population of less than a million, has some of the highest prevalence of diabetes in the world: 16% whereas the global prevalence stands at 8.5% [3–5]. Such high prevalence of T2DM in the Fijian population may be an overture of a stagnant public health-care system since the 1970s and its inability to make robust transition with rapid economic growth and westernization. Furthermore, the *Pacific way of life*, a traditional lifestyle characterized by rural communal active living and healthier home-grown low caloric foods, now clashes with urbanization-related problems, such as unhealthy foods, sedentary jobs, and inability to consolidate tradition with modernization [6].

Reports indicate diseases arising from diabetes are the leading causes of death in the Fijian population and also rank as the highest in causing disabilities in adjusted life years compared to other disease states [7, 8]. Macrovascular complications of cardiac and cerebral diseases are common; diabetic foot amputations have increased from one every twelve hours to now one every eight hours; diabetic sight threatening retinopathy is three times higher in the Fijian diabetic population than compared to other countries; and chronic diabetic nephropathy is the leading cause of end stage kidney disease and death [9–12]. This suggests that the disease burden is getting worse.

There is strong validated evidence that shows that effective management of diabetes decreases macro- and microvascular complications and premature death [13, 14]. The Fijian diabetic practice guideline advocates for management that equals with recommendations from the international health organizations, yet patient outcome data is not reassuring [15, 16].

In Fiji, diabetic patients attend special outpatients' chronic disease clinics (SOPD). Our service of focus is a secondary care hospital based in Labasa, the highest level of health care available for the northern part of Fiji, and it provides care for both newly diagnosed diabetics and also patients with established complications and comorbidities. This clinic is run by general medicine internists (there existed only two board-certified general medicine internists for the northern division—with a population size of 130,000—during the course of this study; there were no endocrinologists). The service of study has at its disposal trained foot care nurses, dieticians, ophthalmologists, and other services that warrant a multidisciplinary team approach (MDT) in the management of chronic illnesses.

We hypothesize that a dysfunction in health-care delivery system at patient and physician contact level may be affecting patient outcomes and thereby accentuating the diabetic disease process. This could be due to first, unavailability of any clinically audited data of the services that is being provided which reflects upon patient care; second, no new initiatives being introduced to identify clinical discordance that links high rates of morbidity and mortality; third, no interlinked platforms that synthesize data from different MDTs that could be used to consolidate clinical decision making; and fourth, the available diabetic practice guidelines that may not address some aspects of diabetic management and may be causing an impediment in clinical management.

Therefore, this service evaluation study, the first of its kind to our knowledge, aims to provide insight into the management of T2DM in a secondary care setting in order to scrutinize and streamline services for a better patient outcome. This paper evaluates (1) patients' glycemic control and its management, (2) use of HbA1c tests as a monitoring tool, (3) patient barriers and remedies in achieving optimal glycemic targets, (4) collaboration of MDT assessment in clinical decision making, and (5) diabetic comorbidities present in patients.

## 2. Method

A retrospective chart review methodology was employed. Data collected are from hard copies of patient records, primarily physician notes and laboratory and diagnostic reports which were derived from the standardized patient records form from which clinical decisions have been made. The online patient records system (PATIS) was also accessed to purposefully extract biodata and verify laboratory test reports.

A clinic load scan of the service was done, and an approximate of 220 patient load per week was identified. Random quota sampling was used to extract patient folders of 10% to 15% of the total patients presenting to the clinic each week. Over a one-month period (August-September 2018), 135 patient folders were collected. These folders were then sifted against the following predefined inclusion and exclusion criteria.

*Inclusion criteria* are as follows: (a) has T2DM, (b) has had T2DM for ≥1-year duration, and (c) has attended at least 2 clinics at this service.

*Exclusion criteria* are as follows: (a) has T1DM, (b) has T2DM and progressed to requiring insulin, (c) has had T2DM for <1-year duration, and (d) has attended only 1 clinic at this service.

Charts of patients that were excluded (n=number); n = nine had progressed to requiring insulin, n = three were taking insulin, n = five had T2DM for <1 year, n = five had attended this clinic once.

A total of 113 charts met the inclusion criteria and were analyzed.

Data collection was done by the principal researcher using a data collection template. The information extracted for evaluation was for three years (August 2015 to September 2018).

Study interventions data included: age, sex, ethnicity, duration of diabetes, initial and final random blood glucose (RBS) levels, HbA1c results and frequency of tests, medications and its dosing, diabetic-related comorbidities, ischemic heart disease (IHD), peripheral vascular disease (PVD), stroke, retinopathy, nephropathy, and neuropathy, evidence of involvement of MDT approach- Dietetic services, Foot care and Ophthalmologic assessments in aiding management. Lastly, data pertaining to patient barriers in achieving optimal glucose control as identified by physician in uncontrolled patients was extracted.

The measures for evaluating optimal treatment management are described in Table 1 which is recommended by the Fijian national diabetes practice guideline [16].

Data analysis was carried out using Microsoft Excel 2016 version. Double entering of information was also done to cross-check for consistency and further analyzed using SPSS version 24 (SPSS Inc., Chicago, IL, USA) statistical software package.

Prior to extraction of data, ethical approval was sought from the hospitals' research and ethics committee and later approved by the National Health and Research Ethics Review Committee, Fiji Islands. All patient data extracted was anonymized.

## 3. Results

### 3.1. Patient Characteristics

A total of 113 patient charts were reviewed. 36% (*n* = 41) were males, and 64% (*n* = 72) were females. Ethnically, the sample comprised 88% (*n* = 99) of Fijians of Indian descent (FID) and 12% (*n* = 14) were Itaukei (ITK). Overall, patients' average age was 62.8 years, the youngest being 34 and the oldest 86 at the time of this study. Mean duration of diabetes was 7.5 years. The minimum duration of T2DM was 1 year, and longest duration was 17 years. Further breakdown of patient characteristic is presented in Table 2.

### 3.2. Random Blood Glycemic Control

The most recent RBS level showed that 17% of the patients had optimal glycemic control, 17% had fair control, and 66% of patients were inadequately controlled of which 28% had RBS of ≥14 mmol/L. The mean glucose level was 12.4 mmol/L for all patients.

When comparing change in entry RBS to final RBS levels, 18% of patients managed to improve their uncontrolled diabetes to controlled levels by a mean glucose reduction of 5.3 mmol/L. However, 11% moved from optimal and fair controlled groups to being poorly controlled. 16% remained in optimal and fair control groups.

**Figure 1:**
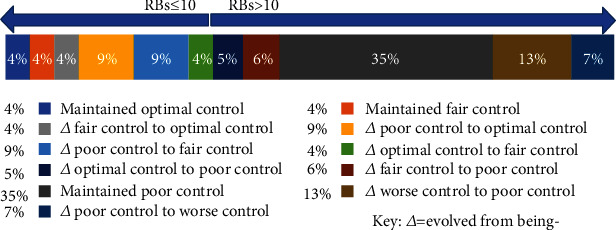
Description of aggregated patients diabetes control over 3 years. Change in RBS control over 3 years.

55% of patients entering the study with uncontrolled states remained uncontrolled of which 35% remained at their initial poor control levels, 7% worsened from poor control to worse control, and 13% who were uncontrolled managed to decrease their mean glucose by 5.8 mmol/L and still remained uncontrolled. This is elicited in Figure 1.

Overall, based on initial RBS results, 27% of patients had RBS ≤10 mmol/L, and 73% of patients had RBS of >10 mmol/L. When comparing initial and final RBS data, relative improvement of 31% was in the optimal group and 21% improvement in the fair controlled group. There was 22% improvement in the group who had RBS of ≥14 mmol/L; however, in 3%, worsening of RBS was seen in the 10-14 mmol/L group.

### 3.3. Glycemic Management

Overall, 2% (*n* = 2) of patients were on diet therapy, 19% took metformin (*n* = 22), 32% were on glipizide (*n* = 36), and 2% (*n* = 2) on glibenclamide as single oral agents. 44% (*n* = 50) were on combination of metformin and glipizide and 1% (*n* = 1) on combination of metformin and glibenclamide.

When evaluating level of dosing with metformin, 45% were at level 1 dosing, 41% at level 2, and 14% at level 3.

With glipizide, 45% were at level 1 of dosing, 22% at level 2, and 33% at level 3. Patients taking glibenclamide were at level 3 dosing. 2% were not on any drugs.

In combination drug consumers, 14% were at level 1 dosing, 25% at level 2, and 61% at level 3 dosing.

In the combination group, 12% (*n* = 6) were on maximum dosage of 2 drugs, and the next intensification will be insulin; 29% (*n* = 15) were at maximal dose of 1 drug, and the next intensification of the other oral drug will result in 2 drugs at maximal dosages.

### 3.4. HbA1c Tests in Clinical Decision Making

Over 3 years, 87 HbA1c tests were conducted on which clinical decisions were made, and the results of 30 tests existed on PATIS, without it being used to make any decisions. 48% of the participants did not have any HbA1c tests done on them. When accommodating for individual patient years at this clinic, analysis showed that 441 tests should have been conducted; however, only 20% of this was done.

Of the 87 tests, 35 tests were done once in individual patients and 20 tests twice, and 4 patients had the test done thrice on them.

For repeat HbA1c tests, 8 were conducted 6 months apart, 11 were repeated a year apart, and 13 tests repeated after a period of 13 months to 2 years.

To evaluate meaningful results of patients whose tests were repeated 6 months apart, 6 decreased their mean HbA1c by 1.52%, and 2 patients increased their mean HbA1c by 1.6%.

### 3.5. Barriers and Remedies in Achieving Glucose Control

For those patients who had RBS of >10 mmol/L (*n* = 75), the main reason identified for being uncontrolled was noncompliance to medications, diet, or exercise in 47%; 4% defaulted clinics; 9% had medication intolerance; and no reason was identified in 40% of patients.

To mitigate for identified issues, 3% of patients were referred to dieticians or diabetes hub center, 80% received SNAP intervention (smoking cessation, good nutrition, no alcohol, and advocating for physical activity) by the attending physician, and 17% did not receive any interventions.

### 3.6. Collaboration of MDT in Clinical Decision Making

Service provided by three MDTs to patients, based on which clinical decisions had been made, was looked at. One patient had grade of retinopathy assessment reported and 99% of patient did not have any clinical data available in relation to diabetic eye disease. 9% of patients were referred to an ophthalmologist over 3 years.

Three percent of participants had documented peripheral neuropathy, and 3% had amputations; however, no foot risk classification was made in 100% of patients. 5% of the patients were referred to the foot clinic for foot assessment.

Patients referred to dietetic services for nutrition advice stood at 8%. 92% did not receive individualized dietetic counselling.

### 3.7. Diabetes-Related Comorbidities

36% of patients had macrovascular diseases and 33% had microvascular complications. Metabolic syndrome features were present in 5%, 27% had hypertension, and 4% had nil comorbidities. 2 patients had dyslipidemia.

The most common disease entity was hypertension present in 77% (*n* = 87) of patients, IHD- second common in 36% (*n* = 41) and chronic kidney disease (CKD) present in 27%. These figures are presented in Table 3. 36% (*n* = 41) of patients had not developed any end organ complications.

Other common diseases present were thyroid diseases, colitis, rheumatic heart disease, valvular heart disease, and gout (15%; *n* = 17).

## 4. Discussion

### 4.1. Critical Analysis of Results

This study, even though small, is the first of its kind to evaluate diabetes care in this secondary level institution. Results indicate T2DM populous with highly uncontrolled blood glucose levels, a high burden of diabetic complications and HCP's accentuating this process by not fully utilizing available resources.

RBS levels used as glycemic monitoring are a valuable and affordable instrument in resource limited settings. Studies have quantified that RBS levels correlate with HbA1c to some extent [17, 18]. However, recognizing that RBS levels does not project accurate HbA1c measurements since patients' blood glucose fluctuates throughout the day, hence, this needs verification through conduction of HbA1c tests so that the patients' overall glucose controls are confirmed and used to guide management targets [18, 19].

Most of the participants in this study did not have HbA1c testing done, and therefore, true definition of control is eluded. For the purpose of conceptualizing appropriate control based on HbA1c percentages, the mathematical estimation calculator derived from the DCCT study, to convert RBS to percentages of HbA1c, was employed [20]. Hence 66% of patients had estimated HbA1c of >7.5%. When comparing the National Diabetes Audit data from the UK, only 34% of T2DM population had HbA1c of >7.5% [21]. Even though this small study does not compare to that of national audits, other smaller observational studies, reflecting real life-effect, have half the number of patients with HbA1c of >7.5% than compared to our participants [22].

In the Fijian health service, there are only three oral antidiabetic drugs for the management of T2DM: metformin and two sulfonylureas, namely, glipizide and glibenclamide [16].

Overtime, pancreatic *β*-cell function declines, and these increase glucose levels in diabetics [23]. Monotherapies commenced are effective initially, but these oral agents are prone to failure overtime requiring add-on of other antidiabetic drug classes to exert euglycemia [24, 25]. Studies [26] have also shown that the combination therapy of metformin and SUs generally tends to deteriorate as early as 6 months after commencement.

In our sample size, for those taking single oral drugs (*n* = 60), 28% of them were at maximal doses and would require add-on therapy in the near future. For those patients on combination therapy (*n* = 51), 41% of them would need intensification to insulin over the next few years.

Theoretically, if we consider initiating insulin based on HbA1c results according to international recommendations, HbA1c >7.5% add-on insulin therapy and HbA1c >10% patient must be on insulin (27); 38% of total patients in this study require insulin in combination with oral drugs; and in 28% of patients, insulin is essential as they have likely attained oral treatment failure [27].

HbA1c tests are the accepted tool used to monitor glycemic control. The validity of these tests as a predictor for micro- and macrovascular complication development, diagnosing new diabetes and overall patient management guidance, is well documented [28].

International guidelines suggest that HbA1c tests should be conducted every 3-6 months; for uncontrolled patients, the frequency is 3 monthly; and those optimally controlled, 6 monthly monitoring is sufficient [29].

A large study in the UK showed that 3 monthly HbA1c testing led to a reduction of HbA1c by 3.8% and the annual testing increased HbA1c by 1.5% [30]. This therefore suggests that the frequency of HbA1c test is an important indicator and tool for achieving optimal glucose targets.

In our study, close to half of the participants did not have any HbA1c test done in the last three years. 31% of patients had HbA1c done once. Variation in HbA1c testing frequency exists in different health-care settings. A study done in Canada [31] showed that 36% of diabetic patients had HbA1c tests done once over 2 years.

However, conducting HbA1c tests comes with its own sets of problems. Resource poor setting as this service runs out of reagents, patients are turned back and eventually stop coming [32]. Physicians may not order tests since this is not clearly defined in the practice guidelines, and patients may not be adherent to this monitoring advice being provided [33].

There are many factors that contribute to poor glycemic control. In our study, nonadherence was identified in 47% of patients. Data available from observational studies indicates that T2DM patients have incidence of nonadherence ranging from 38% to 93% [33–35]. The reasons for nonadherence are many, but the prominent one identified in this study was intolerance to medication. There were 18% (*n* = 20) of the total sample size who were on ≥2 g of generic metformin/day; out of which, only 6% of patients were identified to be intolerable, yet this medication was continued in them.

Data looking at metformin doses and tolerability suggest that doses from 2 to 2.5 g cause gastrointestinal side effects and lose its potential to confer any additional glucose regularity benefits. In turn, this has been linked to high rates of patient nonadherence [36, 37].

In 40% of patients, no barriers to achieving appropriate glycemic control were identified. Evidence suggests that lack of integrated care, clinical inertia amongst HCPs, low patient education levels, and their perceived beliefs about treatment inefficacy cause neglect in management [38].

However, it was worth noting that 80% of the uncontrolled patients received point of contact physician SNAP counselling. Even though this form of vertical counselling has been ongoing for years, patient outcomes have not been improving. Observational studies [39] indicate that individual counselling by physicians is generally not well perceived by patients, and this needs reevaluation in this service.

To help patients achieve optimal control, this service can utilize the help of dieticians and diabetic hub counselling services; however, only 3% of patients were referred. This suggests that physicians are using the “5A” model of behavior change partially where they are “assessing” and “advising” patients but are failing to “assist” and “arrange” the other sequences to enact requirements of behavioral change [40]. Moreover, failure to comprehend that behavior change is necessary in patients; 17% of the uncontrolled group received no form of intervention to help them get on top of their poor glucose control.

Multidisciplinary specialists care and decisions from such interventions help management intensification which has been shown to improve patient outcome through improvement of metabolic control and subsequently prevention of micro- and macrovascular complications and early mortality [41–43].

Data in relation to diabetic retinopathy screening suggests early referral to identify retinopathy, and frequent follow-up screenings prevent progression of diabetic eye disease and sight loss [44]. Foot risk assessments in diabetic patients help prevent foot ulcers and amputations [45]. Nutritional therapy provided by dieticians helps to effectively control blood glucose [46–48].

In our study, 99% of the patients' charts had no information on the status of their retinopathy. No foot risk classifications were made in 100% of patients, and 92% had not received individualized dietary counselling in the preceding 3 years, suggesting that the treating physician has been neglecting the core MDT care processes of diabetic management.

For diabetes-related complications, progression from microvascular to macrovascular is the common sequence of the diabetic disease process [49]. Studies show microvascular complications to be more common than macrovascular complications [22, 49, 50]. In our study, macrovascular complications were more common, and this digressed from the normal pathophysiology. This suggests, since efforts are not made to evaluate microvascular risks, therefore effective intensification of treatment is delayed which potentiates to development of irreversible end organ damage. At this juncture, treatment intensification is commenced, but already the true “time impact” of medical intervention has passed.

### 4.2. Implications of Clinical Practice

From this study, we suspect the following reasons are why diabetes care processes and adequate management of T2DM patient are lacking.

First, the presence of clinical inertia and clinical laxity. Clinical inertia is defined as “recognition of the problem, but failure to act” [51]. Data showed that physicians have at their disposal tools and resources that are not being used effectively. This may be due to unfamiliarity with guidelines and lack of clinical experience as most doctors at this clinic are fresh graduates and training to be medical internists. Moreover, patient numbers to physician ratio are high, and rushed consultations take precedence when these doctors have other ward responsibilities.

Second, although diabetes practice guideline provides advice for most aspects of diabetic care, it eludes recommendations of certain key practice points, such as frequency of conduction of HbA1c tests in uncontrolled patients, how and when to intensify treatment, and when exactly to commence insulin.

Third, since this service does not use any online platform systems for patient note recording, thus, hand-written folders are used. This method of note recording is ineffective in consolidating MDT assessments which happen in isolation.

Fourth, disconnect from patient-centered care and vertical physician-led management are prevalent. Counsellors, patient behavioral support systems, and structured education programs are unavailable. This vertical management weakens physician-patient trust which itself is difficult to foster in a public-funded service, resulting in patients become ignorant of their disease, hence high rates of nonadherence.

Fifth, resource limitations, absence of diabetes specialists, and lack of training opportunities provide deviation of accepted diabetic management. Intensifying diabetic drug management becomes a challenge as a result of fewer choice of drug classes, and trainings are not provided to general practitioners or medical officers on initiating insulin; therefore, the cycle of clinical inertia perpetuates due to the absence of qualified individuals to take the lead to provide advisory role in guiding acquisition of better resources and to capacity build.

Lastly, quality improvement efforts are nonexistence. There are no audits of clinical parameters at local and national levels; hence, the ability to recognize problem areas and develop action plans to mitigate service challenges and implement new methods of tackling and improving diabetes management issues is not comprehended.

## 5. Recommendations

The following recommendations are suggested in improving patient outcome and the quality of health care.

First, detailed in-service training on the diabetic guideline needs to be implemented through continuous professional development initiatives. Junior medical officers need to be aware of practice guidelines, and refresher trainings should be offered to other HCPs.

Second, the service should consider strengthening diabetes care by initiating the diabetology speciality training stream in medicine or appointing a clinical lead in this area.

Third, diabetes practice guidelines need to undergo review and clearly redefine some key practice points. Additionally, the guideline committee needs to include in its recommendations effective tools and strategies for clinical practice.

Fourth, adoption of the alphabet strategy as a tool that links MDT assessments to individual patient case management provides the means for affordable consolidation of diabetic care [52]. This validated tool provides the HCP a horizontal view of different MDT's managements and compels the attending physician to act on them. In addition, this tool takes away the vertical mode of current management autocracy from physicians and advocates for patients to take more ownership of their own disease process and management.

Fifth, use and spend resources more astutely for the benefit of patients. The service should consider sourcing a third of its metformin supply in modified release formulation so that it can cater for those patients that are showing intolerance to generic formulations [53, 54]. In addition, consideration should be made in adding the now affordable generic thiazolidinedione to the formulary, providing an additional step in drug intensification [15, 55], furthermore, realizing the full potential of human resource available (counsellors, dieticians, and diabetes practice nurses) and using their expertise to develop and implement patient education programs.

Lastly, continuous clinical quality improvement measures such as service evaluation and clinical audits need to be adopted and frequently conducted so that problem areas can be identified and improvements can be initiated.

### 5.1. Strengthens and Limitations

The small sample size and potential covariates impinge on making strong accurate associations; however, this study reflects true clinical practice, a real-life situation. Possible limitations include patient chart selection bias resulting in demographic differences because of the sampling method used. Observer bias is likely since the principal researcher collected data independently. Moreover, the data analyzed from this secondary care unit which consults “complicated” diabetic cases; therefore, overreporting of prevalence of comorbidities and more “uncontrolled charts” evaluated is expected. However, our goal was to look at care processes and using glycemic control and end organ complications helped to get this into perspective.

## 6. Conclusion

Our study suggests that majority of the patients attending clinics at this service have a high degree of uncontrolled glycemia and comorbidities. This is essentially due to patients not being provided acceptable integrated multidisciplinary care and physicians lacking knowledge on how to practically incorporate these in decision making in a resource-limited clinical setting.

Strengthening ongoing educational initiatives and using the alphabet strategy as tools to streamline diabetic services are the way forward. Evidence-based service evaluations and audits are needed to validate diabetes guideline development. Furthermore, this will address clinical efficacy and planning for improvement in resource allocation, acquisition, and service provision.

This study should set prudence for services around the country and the national regulatory body to consider larger audits for monitoring and delivering quality, astute clinical care for a resource limited health-care system.

## Figures and Tables

**Table 1:** Measures for evaluating optimal management. Description of optimal diabetic management as per the Fijian diabetes guidelines [16].

Evaluation area	Recommendations as per guidelines
Targets for glycemic control	
*RBS*	
Good control (optimal control)	4-8 mmol/L
Fair control (suboptimal control)	8.1-10 mmol/L
Poor control	>10-14 mmol/L
Worse control	≥14 mmol/L
*HbA1c*	
Good control	<6.5%
Fair control	6.5-7.5%
Poor control	>7.5%

*Glycemic management*	
No ODA	Dietary advice
Single ODA	MTF or SU
Combination ODA	MTF + SU

Dosing of oral drugs	
*Minimum*	Level 1 dosing
Metformin 250 mg-1 g/day	
Glipizide 5-12.5 mg/day	
Glibenclamide 2.5-7.5 mg/day	
*Moderate*	Level 2 dosing
Metformin 1 g-2 g/day	
Glipizide 12.6-20 mg/day	
Glibenclamide 7.6-15 mg/day	
*Maximum*	Level 3 dosing
Metformin >2 g/day	
Glipizide >20 mg/day	
Glibenclamide >15 mg/day	

Combination ODA dosing levels	
Level 1	Minimum dose of 2 drugs
Level 2	Moderate dose of 2 drugs or 1 moderate dose and 1 minimum dose
Level 3	Maximum dose of 2 drugs or 1 maximum dose and the other drug with moderate or minimum dose

Using HbA1c tests	Every 6 months

MDT assessments	(i) Foot assessment referral and foot risks classification-yearly (ii) Eye assessment-yearly (iii) Dietician referral

ODA: oral drug administration; SU: sulphonylurea; MTF: metformin.

**Table 2:** Description of patient characteristics. Patient characteristics (*n* = 113).

*Gender*	
Male	*36% (41)*
Female	*64% (72)*

*Ethnicity*	
FID	*88% (99)*
ITK	*12% (14)*

*Mean age (in years)*	62.8
Patient distribution by age	
≤45	4% (4)
46-56	25% (28)
57-67	35% (40)
68-78	32% (36)
79-89	4% (5)

*Duration of T2DM* (in years)	
Mean	7.5
Patient distribution by duration of T2DM	
≤2	24% (27)
3-5	18% (20)
6-9	24% (27)
10-14	23% (26)
≥15	11% (13)

*Patient distribution by:*	
*First recorded SOPD entry RBS levels*	
4-<8 mmol/L	13% (14)
8.1-10 mmol/L	14% (16)
10.1–<14 mmol/L	37% (42)
≥14 mmol/L	36% (41)
*Last recorded RBS levels at SOPD*	
4- <8 mmol/L	17% (19)
8.1-10 mmol/L	17% (19)
10.1–< 14 mmol/L	38% (43)
≥14 mmol/L	28% (32)

**Table 3:** Description of comorbidities present in the study population. Type 2 diabetes related comorbidities in the study population (*n* = 113).

Comorbidities	*N*
*Macrovascular diseases*	*36% (42)*
IHD	*36% (41)*
Stroke	*4% (5)*
PVD	*1% (1)*
*Concurrent macrovascular diseases*	
IHD + stroke	*4% (4)*
IHD + PVD	*1% (1)*

*Microvascular diseases*	*33% (37)*
CKD	*27% (31)*
Retinopathy	*1% (1)*
Peripheral neuropathy	*3% (3)*
Amputation	*3% (3)*
*Concurrent microvascular diseases*	
Retinopathy + peripheral neuropathy	*1% (1)*

*Metabolic syndrome (hypertension + dyslipidemia)*	*5% (6)*
Hypertension only	*27% (30)*
Hypertension + other disease states	*77% (87)*

*No co-morbidities*	*4% (5)*

Key: IHD: ischemic heart disease; PVD: peripheral vascular disease; CKD: chronic kidney disease.

## Data Availability

The datasets generated and analyzed during the current study are available from the corresponding author on reasonable request.
